# Directly Using Ti_3_C_2_T_x_ MXene for a Solid-Contact Potentiometric pH Sensor toward Wearable Sweat pH Monitoring

**DOI:** 10.3390/membranes13040376

**Published:** 2023-03-25

**Authors:** Rongfeng Liang, Lijie Zhong, Yirong Zhang, Yitian Tang, Meixue Lai, Tingting Han, Wei Wang, Yu Bao, Yingming Ma, Shiyu Gan, Li Niu

**Affiliations:** Guangdong Engineering Technology Research Center for Photoelectric Sensing Materials & Devices, Guangzhou Key Laboratory of Sensing Materials & Devices, Center for Advanced Analytical Science, School of Chemistry and Chemical Engineering, Guangzhou University, Guangzhou 510006, China

**Keywords:** MXene, potentiometric sensor, ion-selective electrodes, wearable sensors

## Abstract

The level of hydrogen ions in sweat is one of the most important physiological indexes for the health state of the human body. As a type of two-dimensional (2D) material, MXene has the advantages of superior electrical conductivity, a large surface area, and rich functional groups on the surface. Herein, we report a type of Ti_3_C_2_T*_x_*-based potentiometric pH sensor for wearable sweat pH analysis. The Ti_3_C_2_T*_x_* was prepared by two etching methods, including a mild LiF/HCl mixture and HF solution, which was directly used as the pH-sensitive materials. Both etched Ti_3_C_2_T*_x_* showed a typical lamellar structure and exhibited enhanced potentiometric pH responses compared with a pristine precursor of Ti_3_AlC_2_. The HF-Ti_3_C_2_T*_x_* disclosed the sensitivities of −43.51 ± 0.53 mV pH^–1^ (pH 1–11) and −42.73 ± 0.61 mV pH^–1^ (pH 11–1). A series of electrochemical tests demonstrated that HF-Ti_3_C_2_T*_x_* exhibited better analytical performances, including sensitivity, selectivity, and reversibility, owing to deep etching. The HF-Ti_3_C_2_T*_x_* was thus further fabricated as a flexible potentiometric pH sensor by virtue of its 2D characteristic. Upon integrating with a solid-contact Ag/AgCl reference electrode, the flexible sensor realized real-time monitoring of pH level in human sweat. The result disclosed a relatively stable pH value of ~6.5 after perspiration, which was consistent with the ex situ sweat pH test. This work offers a type of MXene-based potentiometric pH sensor for wearable sweat pH monitoring.

## 1. Introduction

MXenes, a family of two-dimensional (2D) materials, were discovered by Drexel University in 2011 [1]. The MXenes family is comprised of transition metal carbides, carbonitrides, and nitrides with a general formula of M_n+1_X_n_. M represents transition metals (such as Ti, Mo, Nb, etc.), X represents carbon and/or nitrogen, and T*_x_* represents the functional groups on the surface of the MXene [2]. These materials have been demonstrated to have the advantages of high electrical conductivity, excellent mechanical properties, and abundant surface functional groups. Owing to these characteristics, MXenes have thus been widely used in the fields of catalysis (e.g., electrocatalysis and photocatalysis) [3,4,5], energy storage (e.g., batteries and supercapacitors) [6,7] and sensing (e.g., electrochemical sensors and resistive sensors) [8,9].

The concentration of hydrogen ions in physiological fluids such as sweat, saliva, and urine in the human body is closely related to various physiological diseases [10]. Sweat monitoring is a noninvasive way to record health information in real time [11,12,13,14]. Ion-selective electrodes (ISEs) represent a typical analytical method for the determination of ion concentration. However, traditional liquid-contact ISEs consist of an inner-filling solution, which results in difficulty in integration and miniaturization. The developed solid-contact ISEs (SC-ISEs) overcome this challenge based on a solid-state transduction layer [15,16,17,18,19]. The solid-contact layer plays an important role in ion-to-electron transduction, while the ion-selective membrane (ISM) works as the recognition of target ions. SC-ISEs have been widely used in wearable potentiometric ion sensing due to their miniaturization and integration [20,21,22,23]. The state-of-the-art solid-contact potentiometric pH sensors can be divided into three types [24], i.e., ISM-based, organic polymers (e.g., polyaniline), and metal oxide-based configurations. The ISMs containing hydrogen ions have been used for wearable pH sensors [25,26]. However, with ISMs, costly ionophores, possible water-layer effects, and weak mechanical strength could be rather challenging for their long-term wearable application. Polyaniline (PANI)-based pH sensors are the most-used devices for sweat pH monitoring [22,23,27,28,29,30]. Polyaniline itself has low toxicity, but its byproducts or monomers could cause potential biotoxicity [31]. Metal oxide-based pH sensors have been relatively less applied for wearable sensors [32]. A RuO_2_ [33,34] and IrO_2_ [35,36,37,38] precious metal-based pH sensor discloses excellent performances but is limited due to scarceness, while the non-precious metal oxides are hindered by relatively low sensitivity (e.g., WO_3_) [39,40,41,42].

In this work, the MXene of Ti_3_C_2_T*_x_* was directly employed as a pH-sensitive material to fabricate a solid-contact potentiometric pH sensor. The Ti_3_C_2_T*_x_* was prepared by etching the precursor of Ti_3_AlC_2_. After etching, the Ti_3_C_2_T*_x_* contains surface-abundant functional groups that could be worked as hydrogen ion-sensitive sites. The HF-etched Ti_3_C_2_T*_x_* disclosed a sensitivity up to −43.51 ± 0.53 mV pH^–1^ in a wide range (pH 1–11) and also a reversible response with a sensitivity of −42.73 ± 0.61 mV pH^–1^ (pH 11–1). In particular, a reversible pH response was shown for this material. Based on the flexible characteristic of the D Ti_3_C_2_T*_x_*, it was further integrated into a wearable device with an Ag/AgCl solid reference electrode. The flexible Ti_3_C_2_T***_x_***-based pH sensor was successfully applied for on-body sweat pH monitoring.

## 2. Materials and Methods

### 2.1. Material and Apparatus

Ti_3_AlC_2_ (99%) was purchased from Jilin Yiyi Technology Co., Ltd. (Jilin, China). Hydrofluoric acid (HF, 49%) and silver chloride (AgCl, 99.5%) were purchased from Macklin (Shanghai, China). Lithium fluoride (LiF, 99%), boric acid (99.5%), acetic acid (99.5%), sodium hydroxide (NaOH, 99%), N-methyl-2-pyrrolidone (NMP, >99.0%), and ferric chloride (FeCl_3_, 98%) were obtained from Innochem (Beijing, China). Potassium chloride (KCl, 99.0–100.5%), sodium chloride (NaCl, 99.5%), lithium chloride (LiCl, ≥99%), magnesium chloride hexahydrate (MgCl_2_·6H_2_O, 99.0–102.0%), Nafion solution (5 wt% in lower aliphatic alcohols and water), polyvinyl chloride (PVC, high molecular weight), tetrahydrofuran (THF, ≥99.9%), tridodecylmethyl ammonium chloride (TDMA-Cl, 98%), and bis (2-ethylhexyl) sebacate (DOS, ≥97.0%) were purchased from Sigma-Aldrich (Saint Louis, MO, USA). Potassium tetrakis(pentafluorophenyl)borate (KTPFB, 97%) and ammonium chloride (NH_4_Cl, 99.5%) were purchased from Alfa Aesar (Haverhill, MA, USA). Hydrochloric acid (HCl, 37%), phosphoric acid (H_3_PO_4_, 85%) and sulfuric acid (H_2_SO_4_, 98%) were purchased from Guangzhou Chemical Reagent Factory (Guangzhou, China). All aqueous solutions were prepared with ultrapure water (>18.2 MΩ cm, Milli Q, Darmstadt, Germany). The triacid mixture consisted of 0.04 M phosphate, boric acid and acetic acid. Britton–Robinson buffers (B-R buffer) with different pH were prepared by mixing different volumes of the triacid mixture and 0.2 M sodium hydroxide.

Scanning electron microscopy (SEM) was carried out to examine the morphology and size of the Ti_3_C_2_T_x_ by using the Phenom nano SEM (Phenom Scientific, Eindhoven, The Netherlands). The crystal structure characterization was recorded by X-ray diffraction patterns (XRD) using a Miniflex 600 (Rigaku, Tokyo, Japan) by scanning in the 2θ range of 5−80° with Cu Kα radiation. The valence states of the materials were analyzed by X-ray photoelectron spectroscopy (XPS) using a Thermo Scientific K-Alpha (Thermo Fisher Scientific, Waltham, MA, USA).

### 2.2. Preparation of Ti_3_C_2_T_x_

The Ti_3_C_2_T_x_ was prepared according to a previous report [43]. Typically, MILD-Ti_3_C_2_T_x_ was synthesized by adding 2 g LiF into 40 mL 6 M HCl solution, followed by stirring until the powder was completely dissolved. Then, 1 g of Ti_3_AlC_2_ was slowly added into the above solution (~10 min). The mixture was kept stirred at 35 °C for 24 h. After etching, the sediment was washed with deionized water by centrifugation until the pH value of the supernatant was higher than pH 6. Then, the sediment was dried in a vacuum oven for 12 h to obtain MILD-Ti_3_C_2_T_x_. The HF-Ti_3_C_2_T_x_ was synthesized by slowly adding 1 g Ti_3_AlC_2_ into 20 mL HF solution, followed by stirring for 24 h at 35 °C. After etching, the sediment was washed with deionized water by centrifugation until the pH value of the supernatant was higher than pH 6. Then, the sediment was dried in a vacuum oven for 12 h to obtain the HF-Ti_3_C_2_T_x_.

### 2.3. Fabrication of Ti_3_C_2_T_x_-Based pH Electrodes

pH sensing material inks were obtained by dispersing 10 mg MILD-Ti_3_C_2_T_x_ or 10 mg HF-Ti_3_C_2_T_x_ into 800 μL NMP and 200 μL Nafion solution. Nafion solution was used as a binder to adhere the sensing materials on the surface of the electrode. Glassy carbon electrodes (GCE) with a 5 mm diameter were used as the substrate electrode. The GCE was well-polished and washed separately in deionized water and ethanol, then dried by N_2_ blowing. The Ti_3_C_2_T_x_-based pH electrode was further prepared by depositing 10 μL of the above ink on the GCE, and then the GCE was dried in a vacuum oven at 60 °C for 1 h. The Ti_3_C_2_T_x_-based pH electrode was conditioned in pH = 1 B-R buffer solution for 2 h before use. The purpose of this conditioning step was to promote the proton transport in the Ti_3_C_2_T_x_ sensing material, which is similar to the conditioning step for the solid-contact ion-selective electrodes. The controlled experiment of Ti_3_AlC_2_-based pH electrode followed the same procedure as Ti_3_C_2_T_x_-based pH electrodes.

### 2.4. Fabrication of Flexible pH Sensor

Firstly, a polyethylene terephthalate (PET) membrane (8 cm × 8 cm) was cleaned by ultrasonication in acetone, ethanol, and deionized water, followed by O_2_ plasma treatment for 5 min. The microwell pattern of Ag electrodes was fabricated by the magnetron sputtering deposition technique AJA Orin5 (AJA, Wellesley, MA, USA) with respective 30 nm Cr and 200 nm Ag layers. Then, the electrodes were insulated by spin-coating a thin layer of polydimethylsiloxane (PDMS) and then dried in an oven at 90 °C for 40 min. The Ti_3_C_2_T_x_ working electrode was fabricated on the obtained Ag-coated PET electrode in the same way as the GCE. The reference electrode was fabricated as follows.

### 2.5. Fabrication of Solid Ag/AgCl Reference Electrode

First, the Ag/AgCl electrode was prepared by immersing the Ag-coated PET electrode into 0.3 M FeCl_3_ solution for 10 s for partial oxidation of Ag to AgCl and was cleaned with deionized water. Then, the reference membrane solution was further coated on the Ag/AgCl electrode. The reference membrane solution was prepared by dissolving KTPFB (0.9 wt%), TDMA-Cl (1.1 wt%), DOS (68 wt%) and PVC (30 wt%) in THF. Then, 40.4 mg KCl and 15.3 mg AgCl powder were added into 250 µL of the above solution. In addition to the reference membrane layer, a reference protective layer was further coated. The reference protective layer cocktail was prepared by dissolving 4 g of PVC (33.1 wt%) and DOS (66.9 wt%) in 50 mL THF. The solid Ag/AgCl reference electrode was obtained by depositing 10 µL of reference membrane solution and 20 µL of reference protective layer cocktail, respectively. The dropping time interval of the two solutions was ~12 h.

### 2.6. Electrochemical Measurement Methods

The potentiometric measurements for sensitivity and selectivity were performed by using a multi-channel potentiometer EMF6 (Lawson Lab, Inc) at room temperature based on a two-electrode system. The working electrode was Ti_3_AlC_2_- or Ti_3_C_2_T_x_-modified GCE. The reference electrode was a saturated calomel electrode (SCE). For the flexible pH sensor, a solid Ag/AgCl electrode was used as the reference electrode. The electromotive force (EMF) between the working and reference electrodes was recorded in different pH solutions in the range of pH 1–11. The B-R buffer solution was prepared by mixing 0.04 M three acid and 0.2 M NaOH with a tunable pH range of 2–11. A pH = 1 solution was prepared by diluting concentrated sulfuric acid.

The selectivity was evaluated by two methods. One was the continual addition of interfering ions, and the other was the separation solution method. The former involved adding each interfering ion (10 mM) into a pH 7 buffer solution to record the EMF. The separation solution method involved measuring potentiometric response curves for each interfering ion from 10^–1^ to 10^–5^ M.

For the on-body sweat pH test, the fabricated flexible HF-Ti_3_C_2_T_x_-based pH sensor was worn on the forehead of a healthy male volunteer. A homemade mini-potentiometer with an input resistance of 10^13^ Ω was connected to the sensor. The data during the on-body test was recorded based on a mobile APP. Before the test, the sensor was calibrated by potentiometric tests in B-R buffer solutions (pH, 5–8). After the on-body test, the volunteer ran again, and the sweat was collected, which was tested by a simple pH strip for comparison. In addition, the flexible pH sensor was calibrated again to examine the stability of the sensor.

## 3. Results

### 3.1. Structures and Compositions of Ti_3_C_2_T_x_

**Figure 1:**
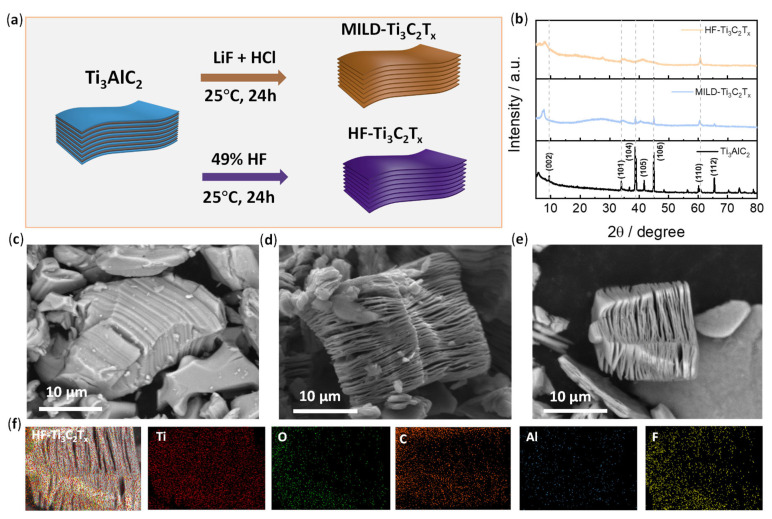
Preparation of Ti_3_C_2_T_x_ and morphologies. (**a**) Schematic illustration of the synthesis of Ti_3_C_2_T_x_ by two etching methods; (**b**) XRD patterns for the Ti_3_AlC_2_, MILD-Ti_3_C_2_T_x_ and HF-Ti_3_C_2_T_x_. (**c**–**e**) SEM images of Ti_3_AlC_2_, MILD-Ti_3_C_2_T_x_ and HF-Ti_3_C_2_T_x_. (**f**) EDS mapping analysis of HF-Ti_3_C_2_T_x_.

The preparation of Ti_3_C_2_T*_x_* was carried out according to the established etching method (see the details in the experimental section). Briefly, two etching reagents of mild LiF/HCl and HF acid were used to exfoliate the pristine Ti_3_AlC_2_ (Figure 1a). After etching, the two products were named MILD-Ti_3_C_2_T*_x_* and HF-Ti_3_C_2_T*_x_*, respectively. Their crystal structures were examined by XRD (Figure 1b). Typical MAX phase (002) planes were observed at nearly 2θ = 9° in the spectrum of Ti_3_AlC_2_. The decreased intensity of the (002) patterns in MILD-Ti_3_C_2_T*_x_* and HF-Ti_3_C_2_T*_x_* was due to expanded interlayer spacing after the successful removal of Al from Ti_3_AlC_2_ [43]. In addition, it was found that the overall crystallinity was significantly weakened after etching, and the characteristic diffraction pattern at 39° for the (104) planes of Ti_3_AlC_2_ disappeared in the XRD spectra of MILD-Ti_3_C_2_T*_x_* and HF-Ti_3_C_2_T*_x_*, suggesting that the Al layer was etched [44].

The morphologies of Ti_3_AlC_2_, MILD-Ti_3_C_2_T*_x_*, and HF-Ti_3_C_2_T*_x_* were examined by scanning electronic microscopy (SEM) (Figure 1c–e). The MAX phase of pristine Ti_3_AlC_2_ exhibits a dense layer structure (Figure 1c). After the etching by LiF/HCl or HF, the structure was exfoliated, thus exhibiting a typical lamellar structure (Figure 1d,e). The interlayer spacing was remarkably expanded. The EDS mapping analysis of representative HF-Ti_3_C_2_T_x_ is presented in Figure 1f. It was found that the Ti, C, O, and F atoms were distributed in the material. However, the content of the Al atoms in MILD-Ti_3_C_2_T_x_ and HF-Ti_3_C_2_T_x_ decreased significantly. Element analysis data shows the atomic content of Al atoms decreased to ~0.5% after etching (Figure S1), which further confirms the successful preparation of Ti_3_C_2_T*_x_*.

**Figure 2:**
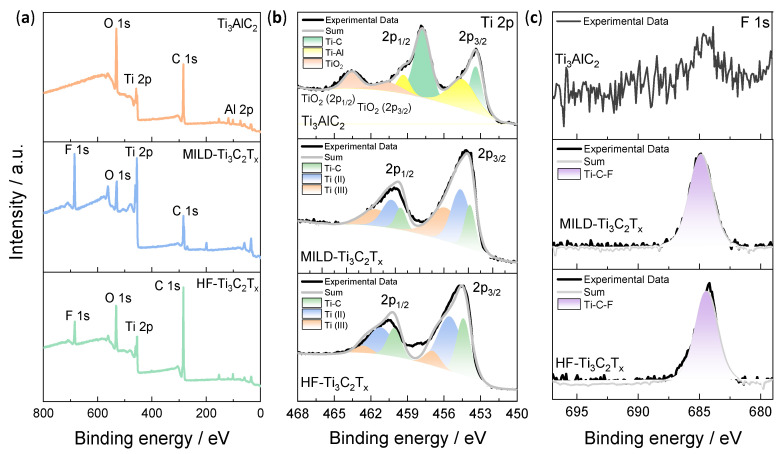
Element compositions and valence states for Ti_3_AlC_2_, MILD-Ti_3_C_2_T_x_ and HF-Ti_3_C_2_T_x_. (**a**) XPS survey spectra of Ti_3_AlC_2_, MILD-Ti_3_C_2_T_x_ and HF-Ti_3_C_2_T_x_. (**b**) XPS spectra of Ti 2p for Ti_3_AlC_2_, MILD-Ti_3_C_2_T_x_ and HF-Ti_3_C_2_T_x_. (**c**) XPS spectra of F 1s for Ti_3_AlC_2_, MILD-Ti_3_C_2_T_x_, and HF-Ti_3_C_2_T_x_.

The elemental compositions and valence states of the Ti_3_AlC_2_, MILD-Ti_3_C_2_T_x_ and HF-Ti_3_C_2_T_x_ were analyzed by X-ray photoelectron spectroscopy (XPS). XPS survey spectra of Ti_3_AlC_2_, MILD-Ti_3_C_2_T_x_, and HF-Ti_3_C_2_T_x_ are shown in Figure 2a. It confirmed the presence of Ti, C, Al and O in the pristine Ti_3_AlC_2_ in which the O element originated from the oxidation of the sample during the long-term storage. After etching, the F element could be clearly distinguished in both MILD- and HF-Ti_3_C_2_T_x_. The high-resolution Ti 2p XPS spectra were further examined in Figure 2b. As shown in the spectrum of Ti_3_AlC_2_, three double peaks were located at 463.6 and 460.5 eV (TiO_2_, 2p_1/2_ and 2p_3/2_), 459.3 and 454.7 eV (Ti-Al, 2p_1/2_ and 2p_3/2_), and 457.8 and 453.4 eV (Ti-C, 2p_1/2_ and 2p_3/2_). As presented in the Ti 2p spectra of MILD-Ti_3_C_2_T_x_ and HF-Ti_3_C_2_T_x_, three double peaks were assigned to Ti-C, Ti(II), and Ti(III), respectively [45]. It was found that the Ti-Al bond of Ti_3_AlC_2_ disappeared, indicating that the Al atom was etched. F 1s XPS spectra further demonstrated the F element introduction after etching (Figure 2c). Overall, the XPS analysis confirmed the elemental compositions of the prepared Ti_3_C_2_T_x,_ and the existence of redox Ti^2+/3+^ could play a role in ion-to-electron transduction.

### 3.2. Potentiometric pH Response

**Figure 3:**
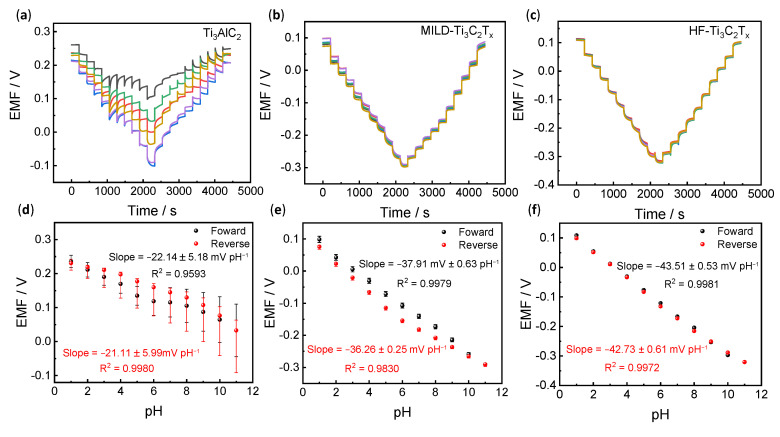
Potentiometric pH responses. (**a**–**c**) Examination of pH reversible responses for Ti_3_AlC_2_, MILD-Ti_3_C_2_T_x_ and HF-Ti_3_C_2_T_x_. All tests have been performed on six individual electrodes as shown in different colors (*n* = 6). (**d**–**f**) pH response calibration curves for the three types of pH electrodes. Corresponding sensitivities for the forward (pH = 1–11) and reverse (pH = 11–1) tests are shown in the Figures.

The above results have identified the structures and compositions of pristine Ti_3_AlC_2_, MILD-Ti_3_C_2_T_x_ and HF-Ti_3_C_2_T_x_. In this section, their potentiometric pH responses were further examined. Figure 2a–c shows the pH reversible responses of the pristine Ti_3_AlC_2_, MILD-Ti_3_C_2_T_x_ and HF-Ti_3_C_2_T_x_ electrodes recorded within the pH range of 1–11. Upon increasing the pH, the electromotive force (EMF) signals of the Ti_3_AlC_2_ electrode show an irregular decrease. It has a poor reversible response and potential stability. The slope for Ti_3_AlC_2_ (−22.14 ± 5.18 mV pH^–1^ between pH 1 and 11) significantly deviates from Nernstian sensitivity. Since Ti_3_AlC_2_ is a raw material without etching, poor electronic conductivity resulted in difficulties in efficient proton-to-electron transduction. In addition, fewer functional groups on the surface of Ti_3_AlC_2_ could cause difficulties for the proton association, leading to low sensitivity. However, the MILD-Ti_3_C_2_T_x_ electrode reveals a reversible response and much-improved reproducibility, and the sensitivity for MILD-Ti_3_C_2_T_x_ increased to −37.91 ± 0.63 and −36.26 ± 0.25 mV pH^−1^ for the forward and reverse pH tests, respectively (Figure 3b,e). HF-Ti_3_C_2_T_x_ further discloses nearly overlapped and reversible pH responses (Figure 3c), and the slope is up to −43.51 ± 0.53 mV pH^−1^ (forward, pH 1–11) and −42.73 ± 0.61 mV pH^−1^ (reverse, pH 11–1) (Figure 3f). These results demonstrated that MILD-Ti_3_C_2_T_x_ and HF-Ti_3_C_2_T_x_, after etching, could efficiently achieve the transduction of protons to electrons. In addition, the introduced functional groups of -OH and -F increased the sites for proton association. Therefore, its pH response sensitivity and reproducibility have been significantly improved.

**Figure 4:**
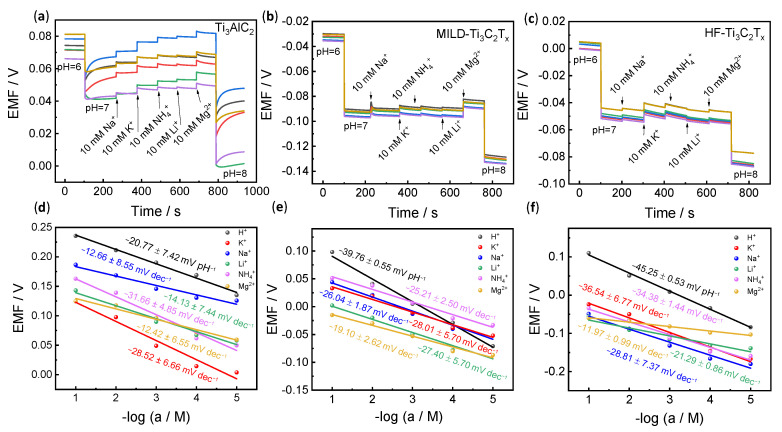
Selectivity evaluation. (**a**–**c**) The selectivity examination of Ti_3_AlC_2_, MILD-Ti_3_C_2_T_x_, and HF-Ti_3_C_2_T_x_ by continually adding interfering ions. (**d**–**f**) The selectivity examination of Ti_3_AlC_2_, MILD-Ti_3_C_2_T_x_, and HF-Ti_3_C_2_T_x_ by separation solution method. All potentiometric tests were performed on six individual electrodes (*n* = 6). The data represent the average values. Corresponding potentiometric response curves are shown in Figures S2–S4.

Selectivity is another important parameter for ion-selective electrodes. Two methods were used to evaluate this parameter. One was the continual addition of interfering ions (Figure 4a–c), and the other was the separation solution method (Figure 4d–f). As shown in Figure 4a, upon changing the pH 6 buffer solution to pH 7, there is an obvious potential response. However, through continually adding 10 mM interfering ions in pH 7 buffer solution, the potential of Ti_3_AlC_2_ changes apparently, while relatively small changes are observed for MILD-Ti_3_C_2_T_x_. In addition, we further evaluated the selectivity using the separation solution method according to Figure 4d–f. When the concentration of the interference ions changes from 10^–1^ to 10^–5^ M, the precursor of Ti_3_AlC_2_ responds to all interference ions, in which the sensitivities for some interfering ions are even higher than the target H^+^ (−20.77 ± 7.42 mV pH^−1^), for example (−28.52 ± 6.66 mV dec^−1^ for K^+^ and −31.66 ± 4.85 mV dec^−1^ for NH_4_^+^) (Figure 4d). The MILD-Ti_3_C_2_T_x_ shows higher sensitivity toward H^+^. However, it should be noted that the potentiometric pH curve (black line, Figure 4e) partly overlaps with interfering ions, and the potential between pH = 3 and 5 is even lower than that of interfering ions (Figure 4e). This result demonstrates that the hydrogen ion selectivity for MILD-Ti_3_C_2_T_x_ remains insufficient. However, the potentiometric pH curve of HF-Ti_3_C_2_T_x_ is above all interfering ions (Figure 4f), which discloses its better selectivity.

The above results demonstrated that the deeply-etched HF-Ti_3_C_2_T_x_ shows the best potentiometric pH response performances compared with MILD-Ti_3_C_2_T_x_ and pristine Ti_3_AlC_2_. The purpose of the etching step is to remove Al atoms in Ti_3_AlC_2_. The precursor of Ti_3_AlC_2_ is nearly an insulator. After etching, the Ti_3_C_2_T_x_ becomes more conductive [43], which is beneficial to proton-to-electron transduction. In addition, plenty of functional groups, such as –OH, –O, and –F, were produced on the surface of Ti_3_C_2_T_x_. These groups could play the role of association sites that exchange with protons and couple with the transition metal redox transition of Ti^3+/2+^ (i.e., proton-coupled-electron transfer), resulting in a potentiometric pH response. Furthermore, the Ti_3_C_2_T_x_ owns an exfoliated structure after etching, which could promote proton transport in the interlayer of Ti_3_C_2_T_x_. We also compared the Ti_3_C_2_T_x_-based pH sensor with literature results (Table 1). It was found that the linear range (pH 1–11) of HF-Ti_3_C_2_T_x_ is better than PANI (pH 4–9) and comparable to metal oxides. Regarding the sensitivity, it is lower than the RuO_2_ and IrO_x_ but competitive to PANI and non-precious metal oxides (e.g., WO_3_ [41] and ZnO [46]). For example, the reported PANI/MXene [30] composite exhibits a sensitivity of −41.91 mV pH^–1^, which is lower than the as-prepared HF-Ti_3_C_2_T_x_ (−43.51 mV pH^–1^). The PANI/LGG-MXene discloses a high sensitivity up to −57.03 mV pH^–1^ but only in a narrow range (pH, 5–9) [47]. In addition, it should be noted that their pH responses originated from PANI. However, our results demonstrate that MXene itself could be directly used as a promising pH-sensitive material. 

### 3.3. Flexible pH Sensor and On-Body Sweat Monitoring

**Figure 5:**
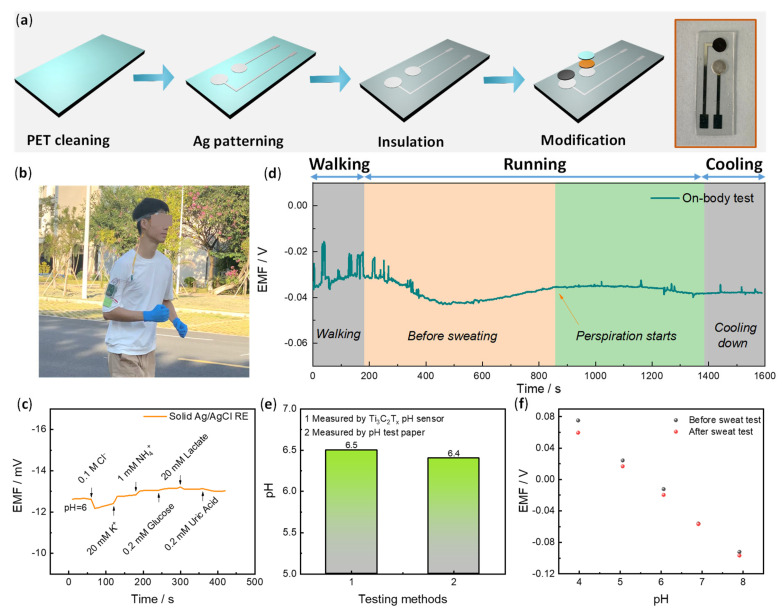
Flexibility pH sensor for on-body sweat pH analysis. (**a**) The schematic fabrication of flexible pH sensor including PET substrate cleaning by O_2_ plasma, Ag patterning by sputter, insulation layer deposition by PDMS, and WE/RE electrode modification by drop casting. The final fabricated flexible pH electrode is shown on the right side. (**b**) A photograph illustrates the on-body test of sweat pH monitoring during outdoor running. (**c**) The ion anti-interference test for the solid Ag/AgCl RE. (**d**) On-body test pH analysis by the device. (**e**) A comparison of sweat pH measured by HF-Ti_3_C_2_T_x_-based pH sensor and pH meter. (**f**) Calibration curves of the HF-Ti_3_C_2_T_x_-based pH sensor before and after sweat test.

The above potentiometric pH response tests have identified that HF-Ti_3_C_2_T_x_ disclosed the best sensitivity, reproducibility and selectivity. In this section, a flexible pH sensor based on HF-Ti_3_C_2_T_x_ was fabricated and applied for on-body sweat pH monitoring. The Ti_3_C_2_T_x_-based pH working electrode (WE) and reference electrode (RE) of Ag/AgCl were designed (Figure 5a–d). The detailed fabrication process was described in the experimental section. Briefly, a flexible polyethylene terephthalate (PET) substrate, after plasma cleaning, was deposited with layers of 30 nm Cr and 200 nm Ag by the magnetron sputtering technique. For the WE, the HF-Ti_3_C_2_T_x_ ink was directly dropped on the conductive Ag substrate. For the RE, the Ag substrate was further transformed to Ag/AgCl by FeCl_3_ oxidation, and the solid electrolyte (polymer-encapsulated KCl) was further coated on the Ag/AgCl (solid RE of Ag/AgCl/KCl). The bending tests for the WE are shown in Figure S5. Upon curving over 60°, nearly overlapped potentials at each pH resulted (Figure S5). These results indicate that the prepared flexible pH sensor could be used for the on-body test.

Finally, on-body pH sweat monitoring was examined (Figure 5b–f). One volunteer ran outdoors with the sensor on the forehead and a homemade mini-potentiometer on the arm (Figure 5b). A sweat belt was used to fix the sensor and direct the sweat to the sensor. The potentiometer contains a Bluetooth module that transports the signal to a cell phone. Before the test, the solid RE of Ag/AgCl was examined toward interference ions and a few representative organic components in sweat. Figure 5c exhibits no apparent potential change upon the addition of these interfering components with a maximum potential fluctuation of less than 1 mV. As shown in Figure 5d, the real-time on-body sweat pH monitoring curve was recorded. After about 14 min, sweat was produced, and the potential showed a relatively steady state. The average pH from 850 to 1250 s was determined to be 6.5 according to the calibration curves. To verify the accuracy of the real-time result, a sweat sample was collected through the same exercise process and was tested with a precision pH paper. A similar value was obtained (pH = 6.4) according to Figure 5e. Additionally, we examined the calibration curves of the sensor after the on-body test (Figure 5f and Figure S6). It was found that there is some degree of potential differences at pH = 4 (~15 mV) and pH = 5 (~8 mV) before and after the on-body test, but this difference is less than 5 mV at pH = 6 to 8. The sweat pH was determined to be around 6.5, so it has no significant effect on the calibration. Overall, the test results indicate reliable on-body pH monitoring in real time.

## 4. Conclusions

In summary, we have developed a Ti_3_C_2_T*_x_*-based wearable solid-contact potentiometric pH sensor for sweat pH monitoring. It has been demonstrated that the deeply etched HF-Ti_3_C_2_T_x_ disclosed the best pH analytical performances compared with the precursor of Ti_3_Al_2_ and MILD-Ti_3_C_2_T*_x_* owing to its high conductivity and abundant surface functional groups for proton association. The HF-Ti_3_C_2_T*_x_*-based pH sensor revealed a sensitivity of −43.51 ± 0.53 mV pH^–1^ (pH 1–11) and −42.73 ± 0.61 mV pH^–1^ (pH 11–1), which is comparable to representative transition-metal oxide-based pH sensors. The sensor also revealed reversible and reproducible characteristics, which was proved by nearly overlapped potentiometric pH response curves upon forward and reverse tests. In addition, the HF-Ti_3_C_2_T*_x_* also showed good selectivity, which was confirmed by both the continual addition method and the separation solution method. Furthermore, this fabricated flexible pH sensing device has realized on-body sweat pH monitoring. The online monitoring pH value is consistent with the ex situ results, suggesting its reliability. The 2D Ti_3_C_2_T*_x_* could be recognized as a new type of potentiometric pH sensor that can be applied as advanced wearable pH devices toward health monitoring.

## Figures and Tables

**Table 1:** The comparison of analytical performances between this work and other reported potentiometric pH sensors.

Materials	Linear pH Range	Sensitivity (mV pH^-1^)	References
PANI/MXene	1–11	−41.91	[30]
RuO_2_	1–13	−49.8 to −59.1	[33]
RuO_2_	2–12	−55	[34]
IrO_x_	2–12	−51.1	[36]
IrO_2_	4–8	−47.54	[38]
WO_3_	1–7	−44.85	[39]
WO_3_/MWCNT	2–12	−41.0	[40]
WO_3_ nanofiber	3–11	−38.5	[41]
ZnO nanorods	4–10	−44.56	[46]
PANI/LGG-MXene	4–9	−57.03	[47]
PANI/CNT	5–9	−45.9	[48]
MILD-Ti_3_C_2_T_x_	1–11	−37.91 ± 0.63	This work
HF-Ti_3_C_2_T_x_	1–11	−43.51 ± 0.53	This work

## Data Availability

The data are available upon reasonable request from the corresponding author.

## Supplementary Materials

The following supporting information can be downloaded at: https://www.mdpi.com/article/10.3390/membranes13040376/s1, Figure S1: Element mapping and compositions for MILD-Ti_3_C_2_T_x_ and HF-Ti_3_C_2_Tx. Figure S2: Potentiometric responses of Ti_3_AlC_2_ electrodes toward a series of interfering ions. Figure S3: Potentiometric responses of MILD-Ti_3_C_2_T_x_ electrodes toward a series of interfering ions. Figure S4: Potentiometric responses of HF-Ti_3_C_2_T_x_ electrodes toward a series of interfering ions. Figure S5: Potential response curves of HF-Ti_3_C_2_T_x_-based pH sensor under normal and bending state. Figure S6: Potential response of HF-Ti_3_C_2_T_x_-based pH sensor before and after sweat test.
