# Supplementary material for: Directly Using Ti3C2Tx MXene for a Solid-Contact Potentiometric pH Sensor toward Wearable Sweat pH Monitoring
Source: Membranes (Basel). 2023 Mar 25;13(4):376. doi: 10.3390/membranes13040376 (PMC10141058; doi:10.3390/membranes13040376)
Supplement: Supplementary file 1 [file membranes-13-00376-s001.zip › membranes-2209606-supplementary.pdf]

membranes

MDPI

Supplementary Materials

Directly Using $Ti_3C_2T_x$ MXene for a Solid-Contact Potentiometric pH Sensor Toward Wearable Sweat pH Monitoring

Rongfeng Liang, Lijie Zhong *, Yirong Zhang, Yitian Tang, Meixue Lai, Tingting Han, Wei Wang, Yu Bao, Yingming Ma, Shiyu Gan, and Li Niu *

Guangdong Engineering Technology Research Center for Photoelectric Sensing Materials & Devices, Guangzhou Key Laboratory of Sensing Materials & Devices, Center for Advanced Analytical Science, School of Chemistry and Chemical Engineering, Guangzhou University, Guangzhou 510006, China
* Correspondence: ccljzhong@gzhu.edu.cn (L.Z.); lniu@gzhu.edu.cn (L.N.)

Citation: Liang, R.; Zhong, L.; Zhang, Y.; Tang, Y.; Lai, M.; Han, T.; Wang, W.; Bao, Y.; Ma, Y.; Gan, S.; et al. Directly Using $Ti_3C_2T_x$ MXene for a Solid-Contact Potentiometric pH Sensor toward Wearable Sweat pH Monitoring. *Membranes* **2023**, *13*, 376. https://doi.org/10.3390/membranes13040376

Academic Editor: Venkatesan Renugopalakrishnan

Received: 25 January 2023
Revised: 6 March 2023
Accepted: 22 March 2023
Published: 25 March 2023

Copyright: © 2023 by the authors. Licensee MDPI, Basel, Switzerland. This article is an open access article distributed under the terms and conditions of the Creative Commons Attribution (CC BY) license (https://creativecommons.org/licenses/by/4.0/).

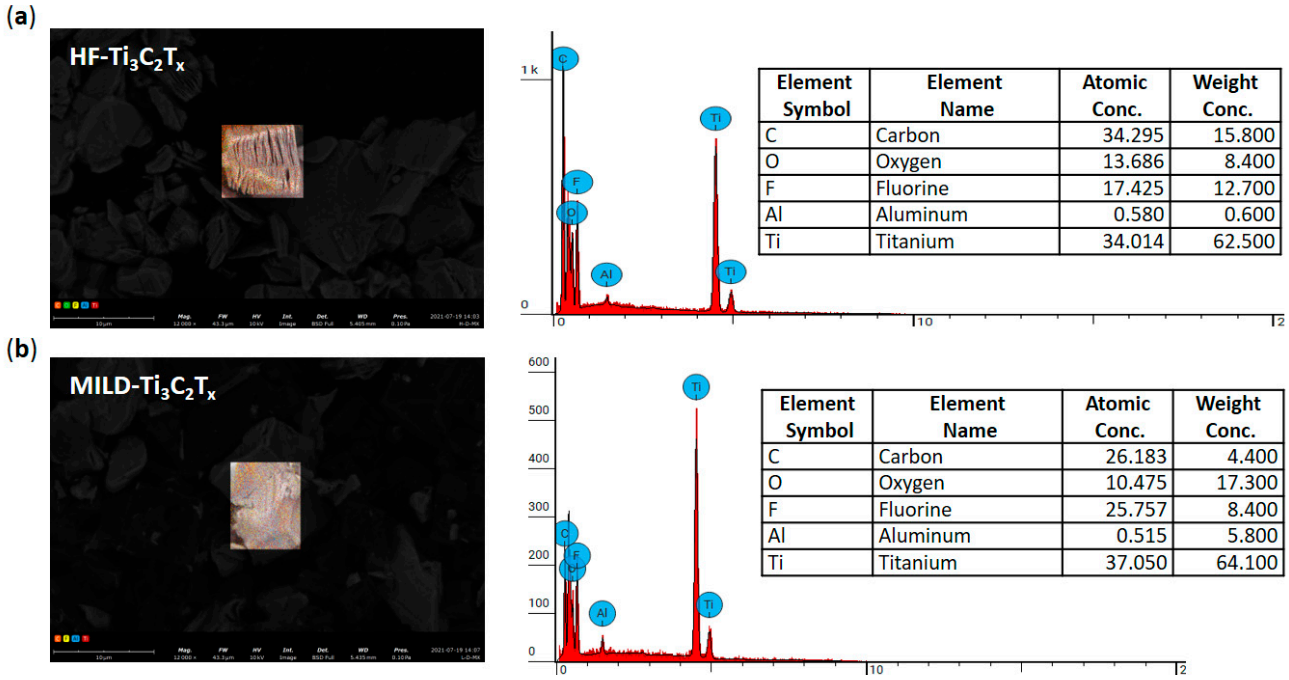

(a)

Element Symbol	Element Name	Atomic Conc.	Weight Conc.
C	Carbon	34.295	15.800
O	Oxygen	13.686	8.400
F	Fluorine	17.425	12.700
Al	Aluminum	0.580	0.600
Ti	Titanium	34.014	62.500

(b)

Element Symbol	Element Name	Atomic Conc.	Weight Conc.
C	Carbon	26.183	4.400
O	Oxygen	10.475	17.300
F	Fluorine	25.757	8.400
Al	Aluminum	0.515	5.800
Ti	Titanium	37.050	64.100

Figure S1. Element mapping and compositions for (a) HF -$Ti_3C_2T_x$ and (b) MILD -$Ti_3C_2T_x$.

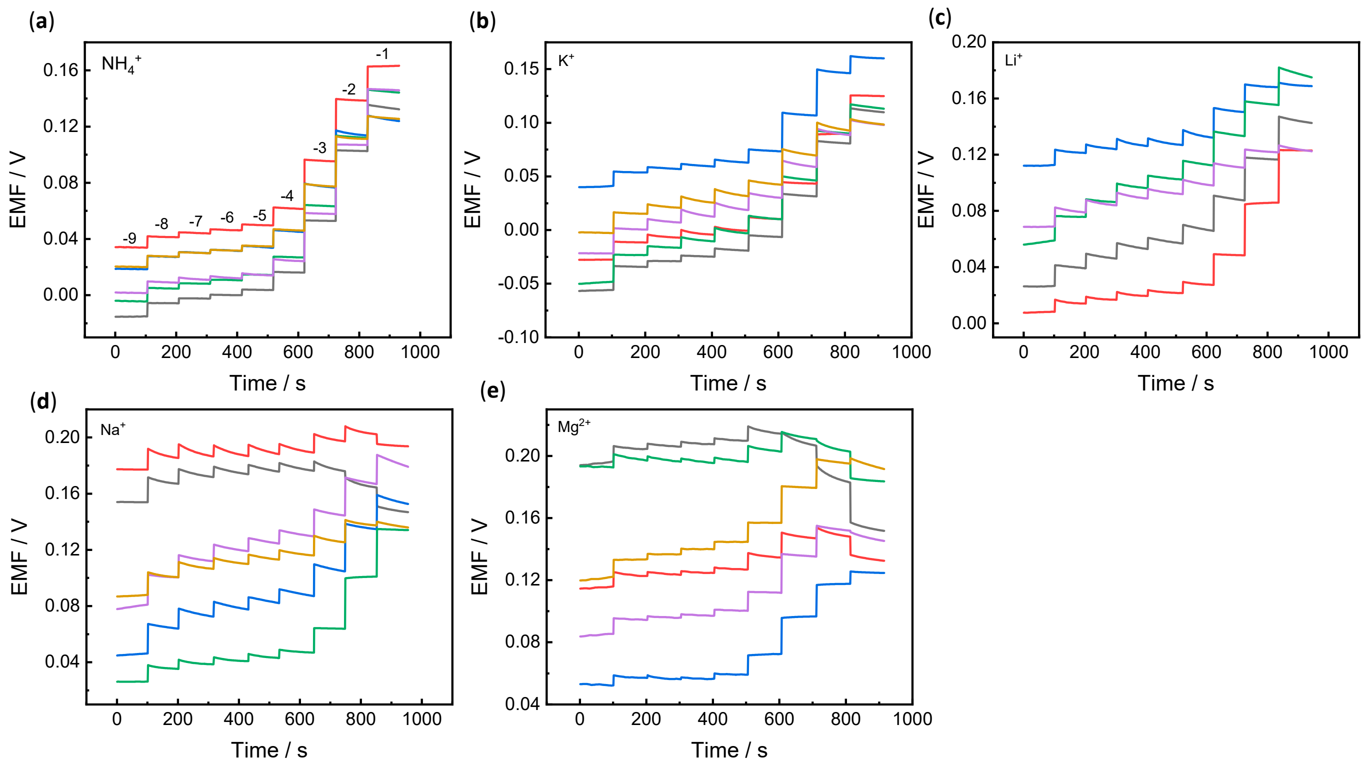

Figure S2. Potentiometric responses of Ti_3AlC_2 electrodes toward a series of interfering ions (a) NH_4^+, (b) K^+, (c) Li^+, (d) Na^+, (e) Mg^{2+} with the concentration from 10^{-9} to 10^{-1} M. The six curves represent tests on six individual electrodes.

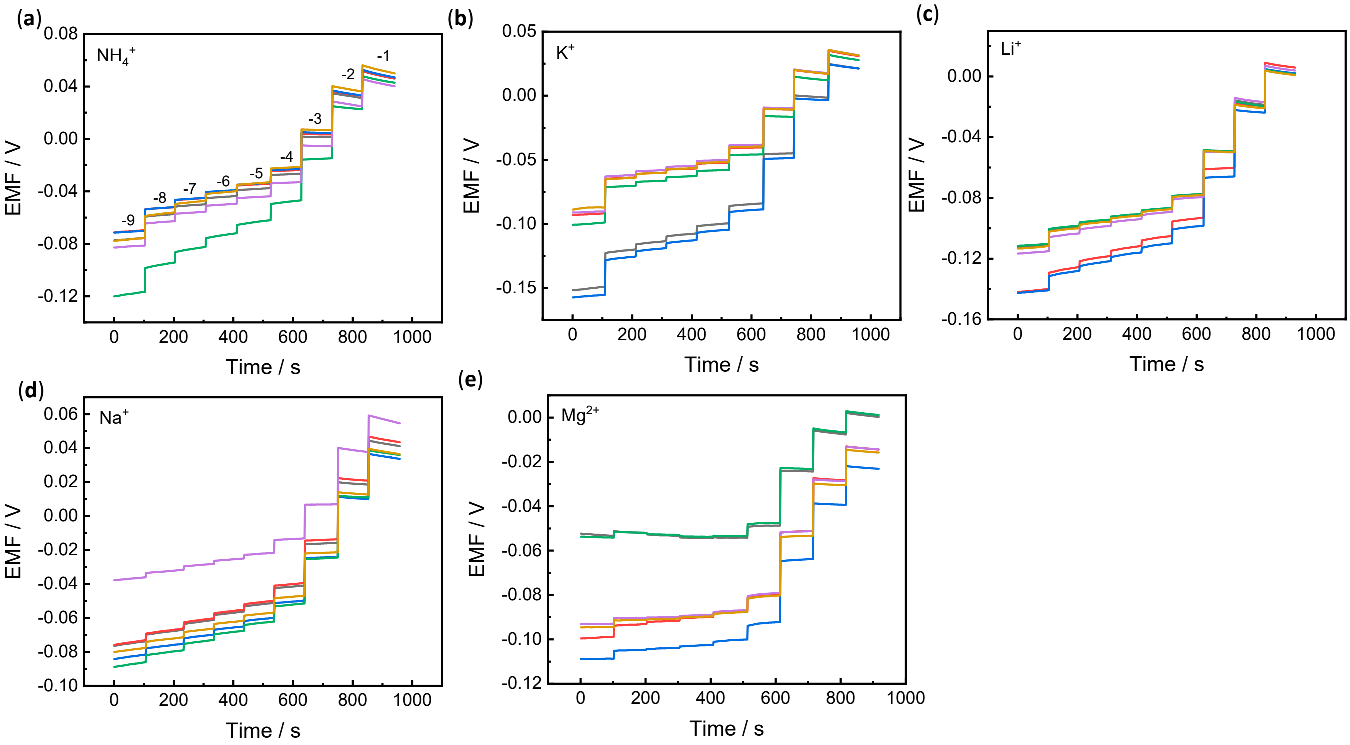

Figure S3. Potentiometric responses of MILD-$Ti_3C_2T_x$ electrodes toward a series of interfering ions (a) NH_4^+, (b) K^+, (c) Li^+, (d) Na^+, (e) Mg^{2+} with the concentration from 10^{-9} to 10^{-1} M. The six curves represent tests on six individual electrodes.

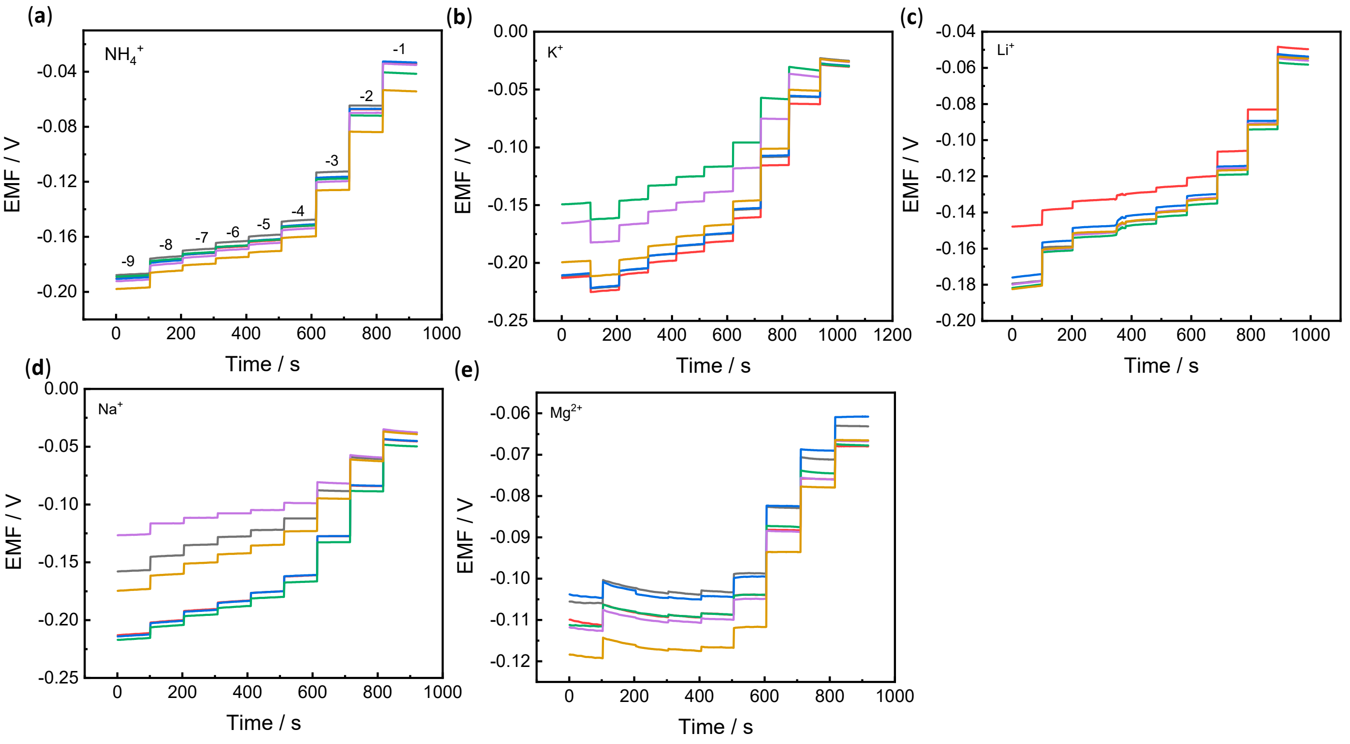

Figure S4. Potentiometric responses of HF-$Ti_3C_2T_x$ electrodes toward a series of interfering ions (a) NH_4^+, (b) K^+, (c) Li^+, (d) Na^+, (e) Mg^{2+} with the concentration from 10^{-9} to 10^{-1} M. The six curves represent tests on six individual electrodes.

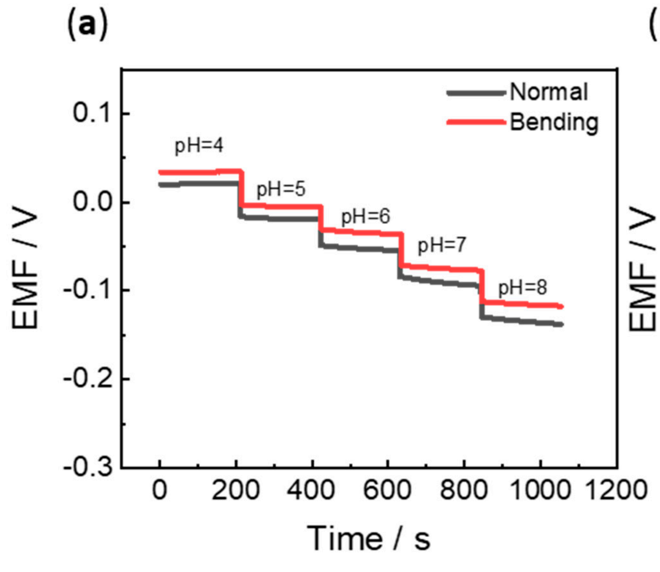

Figure S5. (a) Potential response curves of HF- $Ti_3C_2T_x$ based pH sensor under normal and bending state (over 60°). (b) Corresponding calibration curves under normal and bending states.

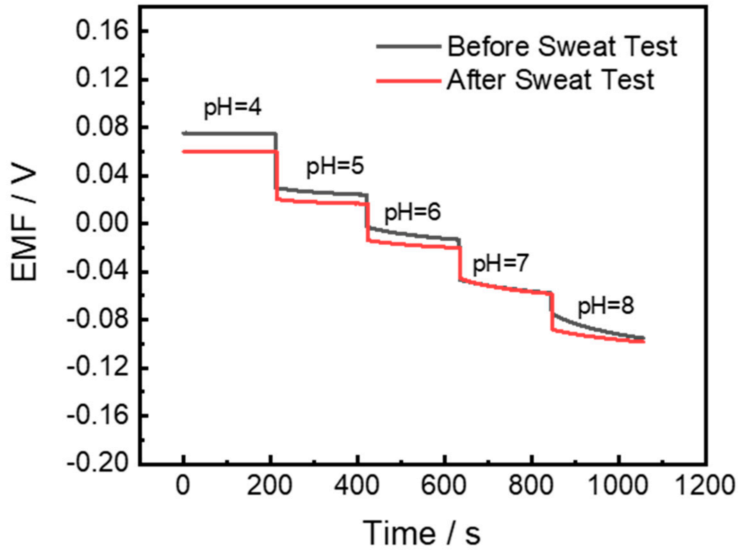

Figure S6. Potential response of HF- $Ti_3C_2T_x$ based pH sensor before and after sweat test.

Disclaimer/Publisher's Note: The statements, opinions and data contained in all publications are solely those of the individual author(s) and contributor(s) and not of MDPI and/or the editor(s). MDPI and/or the editor(s) disclaim responsibility for any injury to people or property resulting from any ideas, methods, instructions or products referred to in the content.
